# [Expression of Concern] Syringin prevents cardiac hypertrophy induced by pressure overload through the attenuation of autophagy

**DOI:** 10.3892/ijmm.2026.5784

**Published:** 2026-03-04

**Authors:** Fangfang Li, Ning Zhang, Qingqing Wu, Yuan Yuan, Zheng Yang, Mengqiao Zhou, Jinxiu Zhu, Qizhu Tang

Int J Mol Med 39: 199-207, 2017; DOI: 10.3892/ijmm.2016.2824

Following the publication of the above paper, a concerned reader has drawn to the Editor's attention that the data shown for the p-AMPKα blots with the RT-qPCR analysis in Fig. 2B on p. 203 are strikingly similar to the ATG7 blots shown in Fig. 4A on p. 205.

The authors were contacted by the Editorial Office to offer an explanation for this apparent anomaly in the presentation of the data in this paper, although up to this time, no response from the authors has been forthcoming. Owing to the fact that the Editorial Office has been made aware of potential issues surrounding the scientific integrity of this paper, we are issuing an Expression of Concern to notify readers of this potential problem while the Editorial Office continues to investigate this matter further.

